# 

**DOI:** 10.1192/bjb.2022.72

**Published:** 2023-10

**Authors:** Chloe Beale

**Affiliations:** Consultant liaison psychiatrist with Homerton Psychological Medicine, Homerton University Hospital, East London NHS Foundation Trust, London, UK. Email: chloebeale@nhs.net

**Figure:**
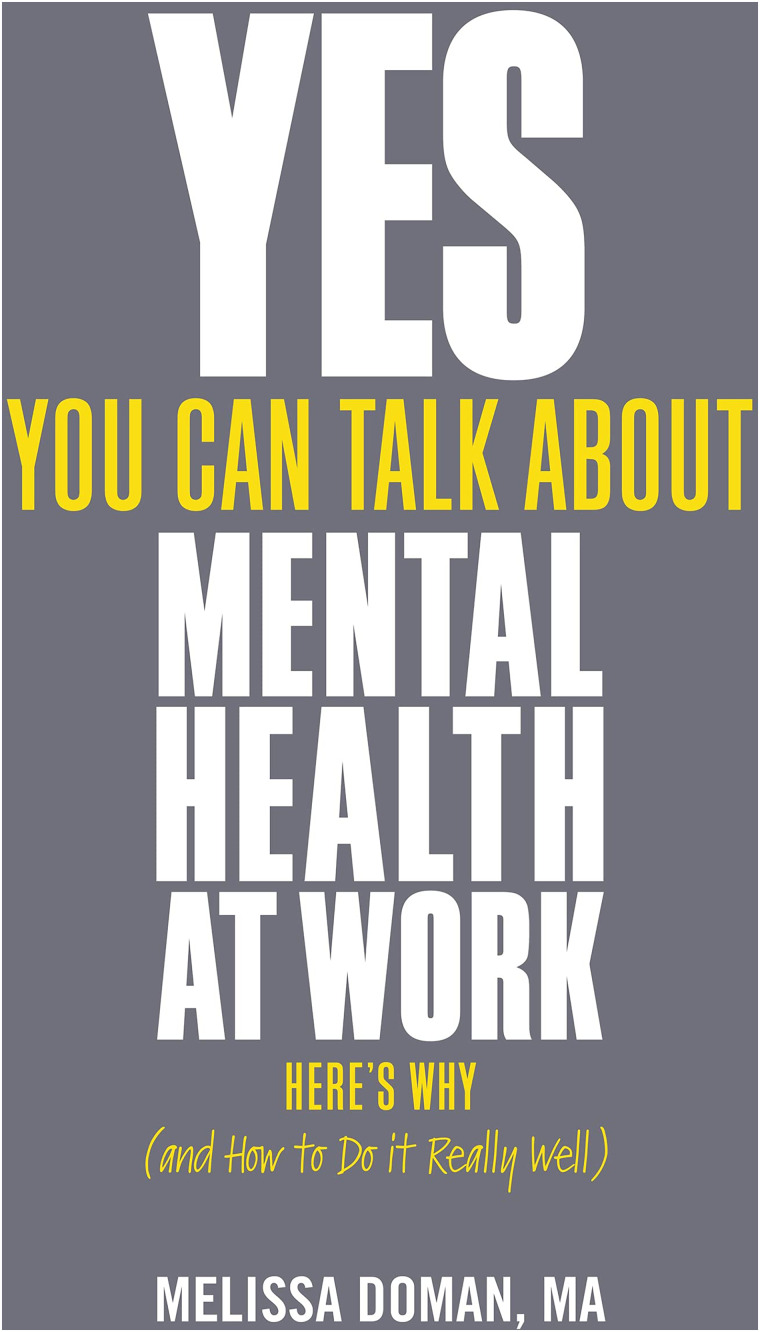

At a time that COVID-19 has brought mental health and well-being at work into sharpened focus, it is appealing to think that managers in various workplaces might take an interest in the subject and be looking for a book to help them.

This book is well-intentioned and may be helpful to those who have no idea where to start in trying to understand mental illness and support their colleagues; it provides some suitable basic explanations of the difference between stress and illness. However, while ostensibly trying to destigmatise mental illness, the author manages to descend into stigma, cliché and oversimplification. For example, she draws a clumsy distinction between ‘high-functioning’ and ‘low-functioning’ illness which, quite apart from being far too simplistic, is othering and effectively tells us ‘we don't need to think about *those* people’. Readers are solemnly warned never to describe someone as ‘suffering’ from mental illness, which strikes me as rather disempowering to those who might feel that mental illness has caused them to suffer a great deal actually. There is a naivety in the clichés (‘it's ok not to be ok!’) and examples of how the tide is turning in terms of stigma (such as Prince Harry speaking about mental health) which I have heard too many times before, though I appreciate I am not the target audience.

Maybe my view is coloured by virtue of working in the NHS, but it strikes me that much of the author's advice is predicated on a supportive workplace where the lower levels of Maslow's hierarchy are already fulfilled. Talking about mental health can feel somewhat pointless when the workplace itself is actively harmful to said mental health, something which is barely touched upon. There is focus on how mental ill health can have an impact on work but relatively little about the reverse.

The section on how to support colleagues and open up conversations will probably be a useful guide to those who have no idea how to broach their concerns and, on balance, this book will probably serve as a helpful aid for thoughtful and well-meaning managers who wish to provide an even more supportive workplace. Much of it does not really seem like specific guidance on mental health, more like common sense advice on how to have conversations and be nice to people.

I was left with the overriding sense that it's ok not to be ok and it's ok to talk about mental illness … as long as it's the right sort of illness.

